# The 7 × 1 Fermi Surface Reconstruction in a Two-dimensional *f* -electron Charge Density Wave System: PrTe_3_

**DOI:** 10.1038/srep30318

**Published:** 2016-07-25

**Authors:** Eunsook Lee, D. H. Kim, Hyun Woo Kim, J. D. Denlinger, Heejung Kim, Junwon Kim, Kyoo Kim, B. I. Min, B. H. Min, Y. S. Kwon, J.-S. Kang

**Affiliations:** 1 Department of Physics, The Catholic University of Korea, Bucheon 14662, Korea; 2 Advanced Light Source (ALS), Lawrence Berkeley Laboratory, Berkeley, CA 12345, USA; 3 Department of Physics, Pohang University of Science and Technology, Pohang, 37673, Korea; 4 MPPC CPM, Pohang University of Science and Technology, Pohang 37673, Korea; 5 Department of Emerging Materials Science, DGIST, Daegu 42988, Korea

## Abstract

The electronic structure of a charge density wave (CDW) system PrTe_3_ and its modulated structure in the CDW phase have been investigated by employing ARPES, XAS, Pr 4 *f* RPES, and first-principles band structure calculation. Pr ions are found to be nearly trivalent, supporting the CDW instability in the metallic Te sheets through partial filling. Finite Pr 4 *f* spectral weight is observed near the Fermi level, suggesting the non-negligible Pr 4 *f* contribution to the CDW formation through the Pr 4 *f* -Te 5*p* hybridization. The two-fold symmetric features in the measured Fermi surface (FS) of PrTe_3_ are explained by the calculated FS for the assumed 7 × 1 CDW supercell formation in Te sheets. The shadow bands and the corresponding very weak FSs are observed, which originate from both the band folding due to the 3D interaction of Te sheets with neighboring Pr-Te layers and that due to the CDW-induced FS reconstruction. The straight vertical FSs are observed along *k_z_*, demonstrating the nearly 2D character for the near-E_F_ states. The observed linear dichroism reveals the in-plane orbital character of the near-E_F_ Te 5*p* states.

The charge density wave (CDW) transition is one of the most interesting phase transitions, which occurs due to the Fermi surface (FS) instability^1^,^2^, like the Peierls transition in one dimension (1D)^3^. The CDW formation is often observed in the low-dimensional systems, and so the CDW phenomenon involves the complicated physics realized in the low-dimensional systems^4^. The CDW state also competes with the exotic ground states, such as magnetic ordering and superconductivity^5^,^6^. It has been controversial whether the FS nesting is the major driving mechanism for the CDW transitions in the two-dimensional (2D) or three-dimensional (3D) systems or whether there exist some other important mechanisms, such as the peak of the real part of the charge susceptibility, 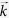-dependent electron-phonon coupling, and electron-electron interactions^7^,^8^,^9^,^10^. In order to understand the nature of the CDW transition, it is crucial to investigate the electronic structure, including the FS of the system. Recent technical progress in angle-resolved photoemission spectroscopy (ARPES) has made it possible to observe the FS topology and the band structures of the solids with the high resolution. Hence, ARPES has become a very good experimental tool for studying the electronic structures of the CDW systems^9^.

The most extensively studied CDW systems by using ARPES are the quasi-2D transition-metal (*M*) chalcogenides (1*T*-*MX*_2_ or 2*H*-*MX*_2_: *X* = S, Se, Te)^6^,^7^,^9^,^10^,^11^, and *R*Te_3_ (*R*: rare-earth ion)^4^,^12^,^13^,^14^,^15^,^16^,^17^,^18^,^19^,^20^,^21^,^22^,^23^. Note, however, that *MX*_2_ and *R*Te_3_ are quite different in that the electrons responsible for the CDW transition are *M* 3*d* electrons in *MX*_2_, whereas those are Te 5*p* electrons in *R*Te_3_. Since the on-site Coulomb interaction (*U*) of Te 5*p* electrons is much weaker than that of *M* 3*d* electrons, the electronic structures of *R*Te_3_ would be simpler than those of *MX*_2_, providing the advantage of studying *R*Te_3_ in investigating the CDW mechanism.

As shown in Fig. 1(a), *R*Te_3_ crystallizes in the quasi-2D layered orthorhombic (close to tetragonal) structure, having three types of Te sites: Te(1), Te(2), and Te(3). Te(2) and Te(3) atoms form the two planar square sheets, which are sandwiched along the *c* axis by the corrugated double layers of *R* and Te(1) atoms^24^. Note that the convention of *a*, *b*, *c* in this work is different from that used in the literature for *R*Te_3_. We have used this different convention to compare the crystal structure of PrTe_3_ with that of *R*Te_2_. Due to the presence of the *R*-Te(1) layer, the 2D unit cell of a Te(2)-Te(3) sheet is doubled (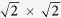), resulting in a smaller Brillouin zone (BZ) (hereafter called as “3D-BZ”), reduced by half and rotated by 45° from the original 2D-BZ of the Te(2)-Te(3) square lattice. Then the shadow bands, which arise from the folding of the bands in the 2D-BZ into the 3D-BZ, are expected to appear in the reduced 3D-BZ. This feature is shown in Fig. 1(b), in which the FS in the outer 2D-BZ is folded into the inner 3D-BZ, to produce the 3D-folded shadow FS (denoted as dotted lines). Consequently, there appear two FSs, one inner (smaller) FS and the other outer (larger) FS. Around Γ_1_ (in the first BZ of the reduced 3D-BZ), the intensity of the former is stronger than that of the latter, while vice versa around Γ_2_ (in the second BZ of the 3D-BZ). In reality, if the interlayer interaction between Te(2)-Te(3) and *R*-Te(1) layers, which causes the band folding, is weak, the electronic structures of Te(2)-Te(3) sheets would keep the 2D nature of the planar square lattice, but would exhibit the 3D-like nature if the interlayer interactions become strong. A stronger interlayer interaction would yield a larger *k*_*z*_ dispersion^25^.

The ionic configuration of *R*Te_3_ is considered to be *R*^3+^Te(1)^2−^Te(2)^0.5−^Te(3)^0.5−^, but this conjecture has not been confirmed experimentally yet. If *R* ions are trivalent (3+), hole carriers are produced in the Te(2)-Te(3) sheets^26^. Then, due to the partial filling, the square nets of two Te(2)-Te(3) planes are easily distorted by the Peierls-like mechanism^27^. Band-structure calculations for a similar CDW system of *R*Te_2_, which has only one partially-filled Te sheet, indicate that the CDW instability occurs from the FS nesting of the Te square sheets in the *ab* plane^24^,^28^,^29^,^30^,^31^.

Despite extensive ARPES and related studies for *R*Te_3_^15^,^16^,^17^,^18^,^19^,^20^,^21^,^22^,^23^, some crucial issues remain to be resolved, listed as following. (i) The modulated structures of *R*Te_3_ in the CDW phase has not been identified. (ii) The valence states of *R* ions in *R*Te_3_ need be determined experimentally. (iii) The effects of the band foldings on the FS of Te(2)-Te(3) sheets, arising both from the interlayer interaction between Te(2)-Te(3) and *R*-Te(1) layers and from the CDW supercell formation, have not been sorted out yet. We have addressed these questions by performing careful measurements of ARPES, Pr 4*d* → 4 *f* resonant photoemission spectroscopy (RPES), and soft X-ray absorption spectroscopy (XAS) for high-quality stoichiometric single crystal of PrTe_3_, and first-principles electronic structure calculations and tight-binding (TB) model calculations for the CDW supercell structure. We have compared the data of PrTe_3_ with those of PrTe_2_. Note that neither ARPES nor the band structure of PrTe_3_ has been reported yet. Owing to the high-quality ARPES data, we were able to perform the state-of-art theoretical analysis so as to identify the modulated structure of PrTe_3_ in the CDW phase.

## Results

Figure 2(a) shows the angle-integrated valence-band PES spectrum of PrTe_3_, compared to that of PrTe_2_, obtained at the Pr 4 *f* resonance energy (*hν* = 124 eV) in Pr 4*d* → 4 *f* RPES. Here we include PrTe_2_ as a reference system that has only one Te sheet in contrast to PrTe_3_ that has two Te sheets. Fig. 2(b,c) compare the Pr 4*d* → 4 *f* on-resonance spectra (*hν* = 124 eV) and those well below resonance (called “off-resonance”). The off-resonance spectra are dominated by the Te 5*p* emissions^32^. The Pr 4 *f* PES spectra exhibit two prominent structures, one well below E_F_ (∼−5 eV) and the other at a low binding energy (BE) (∼−1 eV). The former Pr 4 *f* peak well below E_F_ corresponds to the trivalent Pr 4 *f *^*n*^*c*^*m*^ → 4 *f*^* n*−1^*c*^*m*^ transition (*c*: conduction-band electron, *n* = 2, *m* = 3), and is called as the “bare” Pr 4 *f* peak. The latter Pr 4 *f* structure at a lower BE (∼−1 eV) arises from the hybridization between Pr 4 *f* and conduction-band electrons, corresponding to the 4 *f *^*n*^*c*^*m*−1^ final states^33^,^34^,^35^, and is called as the hybridization peak. This identification for the latter Pr 4 *f* structure is supported by the fact that the features of the low-BE Pr 4 *f* structure resemble those of the off-resonance spectra^32^. Two major differences are observed between PrTe_3_ and PrTe_2_. First, the bare Pr 4 *f* peak in PrTe_3_ is located at a lower BE (∼−4.5 eV) than that in PrTe_2_ (∼−5 eV). Secondly, PrTe_3_ exhibits the finite Pr 4 *f* spectral intensity near E_F_, while PrTe_2_ exhibits the negligible intensity near E_F_. This trend is consistent with the resistivity data, where PrTe_3_ is more metallic that PrTe_2_. Our finding of the finite Pr 4 *f* spectral intensity near E_F_ does not contradict the assumed picture of existing reports^20^,^23^, where the R-Te(1) layers are considered to serve just as a charge reservoir. But the differences in the Pr 4 *f* spectra between PrTe_3_ and PrTe_2_ indicate that the contribution from Pr 4 *f* electrons to the CDW formation is larger in PrTe_3_ than in PrTe_2_. On the other hand, only the Te 5*p* states cross E_F_, but the Pr 4 *f* states do not, and so the Pr 4 *f* contribution would be indirect, *i. e.*, Pr 4 *f* electrons will contribute through the Pr 4 *f*-Te(2)/Te(3) 5*p* hybridization. Hence PrTe_3_ would have the stronger 3D nature than PrTe_2_. It is also notable that the bare trivalent *R*^3+^ 4f states lie in deeper BE as *R* becomes heavier^36^, resulting in the weaker *R* 4 *f*-Te(2)/Te(3) 5*p* hybridization in *R*Te_3_ having heavy *R* elements than in PrTe_3_.

Figure 2(d) shows the Pr 3*d* (*M*-edge) XAS spectrum of PrTe_3_, compared to that of trivalent reference oxide of Pr_2_O_3_. The Pr 3*d* XAS spectrum of PrTe_3_ is found to be very similar to that of trivalent (3 + ) Pr_2_O_3_, indicating that Pr ions are nearly trivalent, having the |*g*〉 ≈ |3*d*^10^4 *f*^ 2^〉 configuration in the ground state. Hence, Fig. 2(d) provides evidence that the valence states of Pr ions are nearly trivalent (Pr^3+^) in PrTe_3_ so that three electrons are donated per Pr ion. This finding supports the previous consensus that the R-Te(1) layers serve as charge reservoirs^20^,^23^, as mentioned above. Two of the donated electrons will fill the Te 5*p* bands of the Pr-Te(1) layers, while one of them fills those of the Te(2)-Te(3) layers. This will result in the *R*^3+^Te(1)^2−^Te(2)^0.5−^Te(3)^0.5−^ configuration. The fully occupied Pr-Te(1) layers become semiconducting. In contrast, the partially filled Te(2)-Te(3) sheets become metallic, which are easily distorted by the the Peierls-like mechanism, as investigated by Papoian and Hoffmann^37^. Hence this finding supports the CDW instability in the Te(2)-Te(3) sheets.

Figure 3 shows the measured FS map of PrTe_3_ over the wide BZ region, in comparison with the calculated FS. The strong and weak FSs in Fig. 3(a), which are reversed in the first and second BZ, respectively, arise partly from the band folding due to the interaction of Te(2)-Te(3) bilayers with neighboring *R*-Te(1) layers. Such features are well established in literature^16^,^19^,^30^ Then, the additional band folding on top of it arises from the CDW supercell structure. The measured FS in Fig. 3(a) appears to be anisotropic: the photoemission intensity around vertical *X* points (±1, 0) is very weak (vanishes), while that around horizontal *X* points (0, ±1) is finite (strong). (Hereafter we call this feature as the “two-fold symmetric” FS feature.) The vanishing photoemission intensity in the FS is expected to arise from the CDW-induced FS reconstruction in the Te(2)-Te(3) sheet. Indeed the absence of the four-fold symmetry has been observed previously in several of RTe_3_^15^,^16^,^19^,^20^, which was then explained by the incommensurate CDW modulation vector^15^,^16^. However, the CDW modulated structures that are responsible for such FSs have not been identified yet. So we have tried to determine the CDW modulated structures, as described in Fig. 3(b,c). As shown in Fig. S1 of Supplementary Information, a reasonably good agreement is found between the measured constant-energy (CE) maps (Fig. S1(a)) and the calculated CE maps for the non-CDW phase of an ideal Te square net (Fig. S1(b)). In contrast, a disagreement is found between the measured and calculated FSs. Such a disagreement comes from the fact that the calculated FS is obtained for the non-CDW phase of PrTe_3_, the structure of which is almost tetragonal (orthorhombic strictly). The two-fold symmetric FS of PrTe_3_ makes a contrast with the four-fold symmetric FS of PrTe_2_, shown in Fig. 3(d).

For the CDW-distorted structure of PrTe_3_, the 7 × 1 structure has been once suggested to be a candidate in view of the observed 7-fold supercell structure in electron diffraction experiments for *R*Te_3_^38^,^39^. We would like to note, however that it is still under debate whether the CDW is uniformly incommensurate or locally commensurate within domains (*i.e.*, discommensurate)^40^. There are two competing schools of thoughts regarding the lattice modulation in the CDW state. One school^38^,^39^ suggested the oligomer-type distortion of the Te(2)-Te(3) atoms in the CDW state, and argued that the CDW is discommensurate. The other school^13^,^14^ reported the long-range order arising from a smooth sinusoidal modulation of the Te-Te bonds (Te-Te dimers) and suggested a single incommensurate *q*_*CDW*_. They argued that the CDW is uniformly incommensurate, which fits well with the FS nesting and Peierls-like distortion, but they did not provide the information on the distortion patterns of Te(2)-Te(3) atoms. Both schools present the similar *q*_*CDW*_ but for physically different reasons, *i.e.,* 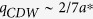. Hence, as a candidate CDW structure, we have considered the 7 × 1 supercell structure, which is large enough to take into account the local distortions of Te(2)-Te(3) atoms for the given 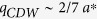.

Figure 3(c) shows the calculated FS of PrTe_3_, obtained from the TB model calculation for the assumed CDW-distorted 7 × 1 supercell structure shown in Fig. 3(b). In order to describe the 7 × 1 super-structure of PrTe_3_, we have first constructed a simple model TB Hamiltonian with the TB hopping parameters for the effective *p*_*x*_, *p*_*y*_ orbitals of Te atoms in the Te bilayer (Te sheets). To get the FS in Fig. 3(c), we have obtained the unfolded band structure by constructing the 7 × 1 Hamiltonian, diagonalizing it, and converting the Bloch factors into those of the cubic cell for the effective *p*_*x*_/*p*_*y*_ orbitals, which are localized in each atomic position. As shown in Fig. 3(c), new features are observed near the (0, ±1) points in the FS for the 7 × 1 structure, while gaps open at (±1, 0), resulting in the mirror symmetry, in contrast to the non-CDW FS of PrTe_3_ (see Fig. S1(b) for the calculated non-CDW FS map.) Remarkably good agreement is found between the calculated FS for the assumed 7 × 1 supercell and the measured FS, indicating that the two-fold symmetric FS in PrTe_3_ arises from the 7 × 1 modulated structure formation in the CDW phase.

Encouraged by the success in describing the experimental FS by combining the TB model and the unfolding technique^41^, we have performed the first-principles DFT calculation for a candidate CDW supercell of 7 × 1 × 1 and then unfolded the calculated band structures. In the DFT calculations, Pr 4 *f* electrons are treated as core electrons. Since the detailed structural data are not available in the literature, we have assumed both ferro-distortion and antiferro-distortion between the two layers in the bilayer, and then relaxed the 7 × 1 × 1 supercell structure by using the VASP code. As for the relaxation calculation, we first assumed local distortions as presented in Fig. 3(b) and then let the system relax around this assumed structure. Interestingly, the shapes of Te-Te bondings (trimers and tetramers), as shown in Fig. 3(b), are maintained with the short and long Te-Te bond lengths being 3.071 \AA and 3.084 \AA, respectively, which are comparable to that (3.077 &Aring;) in the non-CDW phase. This result indicates that the 7 × 1 × 1 structure assumed in Fig. 3(b) is at least a metastable structure. It is also intriguing to note that the relaxed atomic positions maintaining oligomer patterns are described qualitatively well by the sinusoidal function with *q* = 2/7a^*^ (see Fig. S2 of Supplementary Information), suggesting that the CDW structure of PrTe_3_ can be described by either oligomers or sinusoidal distortions. Therefore, we think that the assumed 7 × 1 × 1 supercell structure is close to the correct CDW structure of PrTe_3_, as shown later in the comparison between the DFT-calculated band structures and ARPES. The energy difference between the ferro- and antiferro-cases is found to be negligibly small. Hence we have taken the antiferro-distorted structure for the DFT calculation.

The unfolded band structures calculated for the 1 × 1 × 1 unit cell and the 7 × 1 × 1 CDW-modulated supercell are shown in Fig. 4(a,b), respectively. Red guide lines in Fig. 4(a) represent the TB band structures for the Te square net. The red-, blue-, and green-colored arrows indicate the main band, the 3D-folded shadow band, and the CDW-folded shadow bands, respectively. The black-colored arrow in Fig. 4(b) indicates the calculated CDW gap. These results confirm the reproduction of the intensity differences between the first and second BZs, as observed in the measured ARPES.

The opening of the 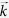-dependent CDW gaps revealed in the FS are expected to appear in the energy distribution curves (EDCs) as the vanishing spectral intensity near E_F_ at certain 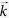 points in the momentum space. Figure 5(a) shows the stack of EDCs of PrTe_3_ for the near-E_F_ region along the diamond-shaped inner FS (along red dots, shown in the inset of Fig. 5(b)). In the EDC stacks, each EDC curve is shifted vertically. Some EDCs, selected from Fig. 5(a), are shown in Fig. 5(b). Guidelines (red lines) are superposed on these selected EDCs to show the 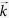-dependent movement of the peak positions and the accompanying intensity modulation near E_F_ (*I*(E_F_)) better. The pronounced movement of the peak positions and the modulation of *I*(E_F_) at different 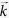 points reflect the opening of the anisotropic energy gap, *i.e.* the 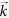-dependent gap Δ_*g*_(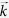) in PrTe_3_. These features are consistent with the findings of the measured FS (Fig. 3(a)).

**Figure 1:**
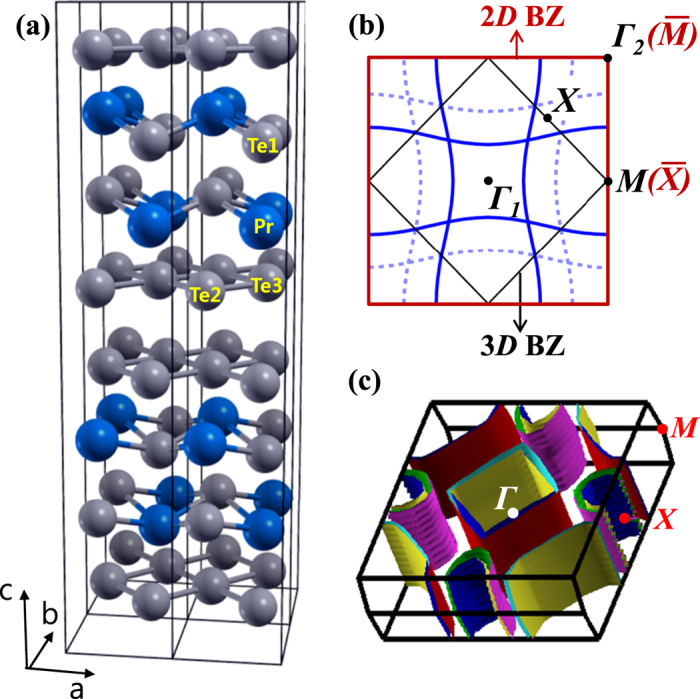
Crystal structure and the 2D/3D-Brillouin zones (BZs) of PrTe_3_. (**a**) Orthorhombic structure of PrTe_3_. (**b**) 2D Fermi surface for the undistorted (non-CDW) phase of PrTe_3_ (denoted as the blue-solid lines). In orthorhombic PrTe_3_ of (**a**), the unit cell of Te(2)-Te(3) square net is doubled. So the BZ of Te(2)-Te(3) sheets (the outer larger square, which we call as the 2D-BZ) is reduced to half and rotated by 45° (the inner smaller square, which we call as the 3D-BZ). The FS in the 2D-BZ is folded into the reduced 3D-BZ, to produce the folded-shadow FS, denoted as dotted lines. (**c**) 3D Fermi surface in the 3D-BZ of the undistorted PrTe_3_.

**Figure 2:**
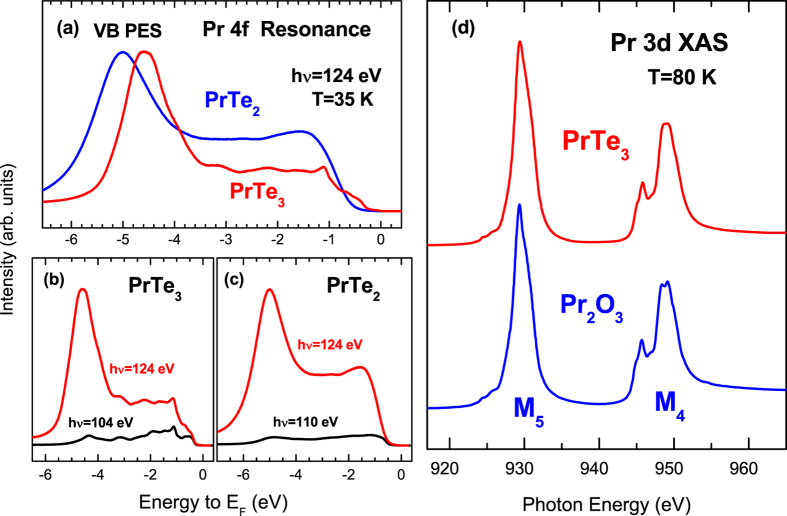
Pr 4 *f* RPES and Pr 3*d* XAS spectra of PrTe_3_. (**a**) Angle-integrated valence-band Pr 4*d* → 4 *f* resonant photoemission spectroscopy (RPES) spectrum of PrTe_3_, in comparison with that of PrTe_2_. Both data were obtained at the Pr 4 *f* resonance energy (*hν* = 124 eV). (**b**) Comparison of the Pr 4*d* → 4 *f* RPES spectra of PrTe_3_, obtained with *hν* = 124 eV (on-resonance) and *hν* = 104 eV (off the resonance), respectively. (**c**) Similarly for PrTe_2_, obtained with *hν* = 124 eV (on-resonance) and *hν* = 110 eV (off the resonance), respectively. The PES spectra were obtained at *T* = 35 K and were normalized to the incident photon flux. (**d**) Comparison of the Pr 3*d* XAS spectrum of PrTe_3_ with that of Pr_2_O_3_, obtained at *T* = 80 K.

Figure 6(a) compares the DFT-calculated band structures of PrTe_3_ having the 7 × 1 × 1 CDW-modulated supercell with the measured ARPES band structures, which are attached as the mirror images. The calculated band structures are unfolded into the 2D-BZ (see Fig. 1(b)) by utilizing the band-unfolding scheme^41^, so that the shadow bands are separated out from the main bands. Here Γ_1_ and Γ_2_ represent the Γ points in the first BZ and the second BZ (in the 3D-BZ notation), respectively. The left-side ARPES is that for the first BZ and the right-side ARPES is that for the second BZ. These ARPES image plots are the average of those obtained with the linear horizontal (LH) and linear vertical (LV) polarizations (see Fig. 7 below). As described above in Fig. 4, the dark and light colors in the calculated band structures denote the main bands and the shadow bands (both the 3D-folded shadow bands and the CDW-folded shadow bands), respectively. Figure 6(a) reveals that, with ∼−0.3 eV shift of the calculated E_F_, the measured ARPES and the calculated band structures agree remarkably well in the dispersive band features and the energy positions. Further, the effect of the band folding, observed in ARPES, also agrees very well with that in the calculations. Thus these findings clearly indicates that the modulated structure of PrTe_3_ in the CDW phase would be close to a 7 × 1 × 1 supercell structure.

Figure 6(b) shows the ARPES image plots near E_F_ for both the first BZ (left) and the second BZ (right) separately. Both data were obtained with the LH polarization. Figure 6(b) reveals the following features. First, the bands crossing through E_F_ are observed clearly, which are responsible for determining the FS at the corresponding 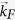 values. The E_F_-crossing positions in ARPES agree with those in the calculated bands for the non-CDW phase of PrTe_3_ (see Fig. 6(a)) very well, which have mainly the Te(2)-Te(3) in-plane 5*p* character. Two crossing bands are observed along Γ*M*, one near 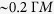 (marked with a red arrow in (b)) and the other at 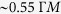 (marked with a blue arrow in (b)). They produce the inner FS and outer FS, respectively, as shown in Fig. 1(b). Note that the spectral intensities of these two crossing bands in the first BZ (left) become reversed in the second BZ (right). Hence Fig. 6 provides evidence for the band folding due to the interlayer interaction of Te(2)-Te(3) layers with Pr-Te(1) layers in PrTe_3_.

In contrast to the previous ARPES work on *R*Te_3_ (*R* = Ce, Tb, Y)^20^, the bilayer splitting is not clearly observed in our near-E_F_ ARPES image plots in the Γ plane within the experimental resolution. Considering the energy and *k*-resolution employed in this work (Δ*E* ≲ 40 meV and 
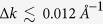
 (*i. e.*, 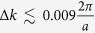 along Γ*M*)), this observation suggests that the size of the bilayer splitting near E_F_ is less than the above experimental resolutions in PrTe_3_. In the previous ARPES work on *R*Te_3_ (*R* = Ce, Tb, Y)^20^, quite a large bilayer splitting was reported along Γ*X* (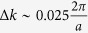 and Δ*E* ≥ 0.1 eV). Our calculations for PrTe_3_, with the spin-orbit coupling (SOC) included, reveal that the size of the bilayer splitting near E_F_ is ∼0.1 eV. But, due to the very dispersive band nature, the splitting of these bands corresponds to 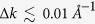 and so it is hardly seen even in the calculated band structures. The difficulty in resolving the bilayer splitting in ARPES is expected to be related to such a very dispersive band nature.

Figure 7 shows the effects of linear dichroism (LD) and circular dichroism (CD) in the measured ARPES of PrTe_3_. Figure 7(a–c) show the measured ARPES image plots of PrTe_3_ along *M*Γ*M* (in the 1st BZ), obtained with two different polarizations of LH and LV polarizations, and the difference between them, respectively. The schematic drawings on the left show the configurations for LH and LV polarizations. In the LH configuration, the electric field vector 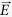 e of the incident photons has a perpendicular component to the sample surface, while 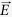 is always parallel to the sample surface in the LV configuration. The difference between LH and LV is defined as linear dichroism (LD) LD ≡ LH-LV. Similarly, Fig. 7(d–f) show the measured ARPES image plots of PrTe_3_, obtained with right circular (RC) and left circular (LC) polarizations, and the difference between them, respectively. The difference between RC and LC is defined as circular dichroism (CD) CD ≡ RC-LC.

The dichroic ARPES image plots for PrTe_3_ reveal several features. (i) Very dispersive bands are observed in ARPES of PrTe_3_ and the large-energy-scale band structures of PrTe_3_ are very similar to those of *R*Te_2_ (*R* = Ce, Pr)^30^,^42^. (ii) The effects of both the LD and CD are observed clearly. The states at −1 eV 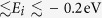 near Γ exhibit the stronger intensity with the LH polarization than with the LV polarization, while the nearly straight bands near *M* from ∼ −4 eV below exhibit the opposite trend. The observed LD in ARPES implies that the orbitals around −0.5 eV and −4 eV below are ordered mainly along the *c* axis and in the *ab* plane, respectively. A closer look at the ARPES near E_F_ shows that, similarly as in PrTe_2_^42^, the E_F_-crossing bands exhibit the stronger intensity with the LV polarization than with the LH polarization. As described above, 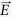 of the incident photons is nearly perpendicular to and parallel to the sample surface with the LH and LV polarizations, respectively (see Fig. 7 Left). Therefore the observation of the stronger intensity with the LV polarization implies that the E_F_-crossing orbitals are ordered mainly parallel to the sample surface, *i. e.*, in the *ab* plane rather than along the *c* axis.

The effect of CD (CD ≡ RC-LC) is more complicated than that of LD. The E_F_-crossing states along (110) have a stronger RC character while those along (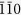) have a stronger LC character. The states at ∼−2 eV show the opposite CD effect. It has been reported that the CD effect arises from the experimental geometry, namely, the experimental handedness^43^. On the other hand, there was also a proposal that the CD arises from the local orbital angular momentum of a system^44^. Hence, there is no general theory yet, which explains the CD effects observed in various systems. If the observed CD has the intrinsic origin partly, the local orbital angular momentum of the Te 5*p* states would be finite in the CDW states of PrTe_3_. However, the nonmagnetic CDW states of *R*Te_3_ seem to refute this idea. Therefore, further theoretical analysis is required to elucidate the origin and the physical meaning of the CD effect in PrTe_3_.

Figure 8(a,b) show the *k*_*x*_ − *k*_*z*_ plane in the BZ of PrTe_3_, for which the *hν*-map was obtained, and the calculated FS for PrTe_3_, respectively. Figure 8(c) shows the measured Fermi-edge *hν* (*k*_*z*_) map^25^ between 80–120 eV with the horizontal direction parallel to (100). Hence Fig. 8(c) represents the measured FS for the BZ of *X*Γ*X*-*RZR*, as shown in Fig. 8(a). The measured *k*_*x*_ − *k*_*z*_ (*hν*) map for the E_F_-crossing states in PrTe_3_ exhibits the straight vertical dispersions along *k*_*z*_, indicating that they have the nearly 2D-like character, and that the interaction between Pr-Te(1) layer and Te(2)-Te(3) layers does not have a crucial contribution to the electronic states responsible for the CDW formation in PrTe_3_. The calculated FS of PrTe_3_ in Fig. 8(b) also shows the straight vertical dispersions along *k*_*z*_. However, an extra lens-like FS, which is present in the calculated FS (Fig. 8(b)), is not observed in the measured FS (Fig. 8(c)). The existence of this lens-like FS need be checked more carefully.

Finally, it is worthwhile to address an important issue in *R*Te_3_, namely, why there exist two-step vs single-step CDW transitions depending on the *R* elements. As mentioned above, *R*Te_3_ has a nearly tetragonal structure, and so there exist two competing FS nesting vectors, *q*_1−*CDW*_ and *q*_2−*CDW*_, along a^*^ and b^*^ directions, respectively. But, due to the lattice strain energy, one direction is thought to be selected first for the CDW transition^45^. However, for heavy *R*Te_3_ having smaller volumes, the FS nesting is far more imperfect than for light *R*Te_3_ having larger volumes, and so there still remains the possibility of the second CDW transition to occur even after the first CDW transition. This would be the reason why there exist two-step transitions for *R* = Dy-Tm while only single-step transitions for *R* = Ce-Tb. These features, together with the reduced densities of states at E_F_ for heavy *R*Te_3_, are also consistent with the reduced first *T*_*CDW*_ for heavy *R*Te_3_. This idea deserves to be checked more systematically further in future.

## Conclusion

**Figure 3:**
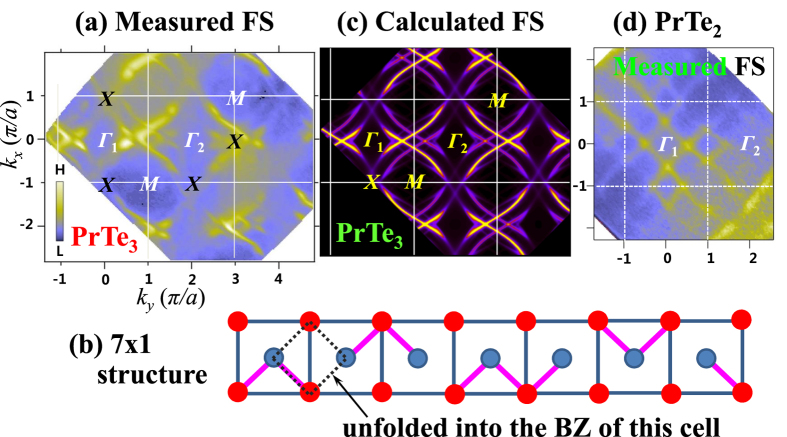
Comparison of the measured FS and the calculated FS for the CDW-distorted PrTe_3_. (**a**) The measured FS map of PrTe_3_ over the wide BZ region, obtained with *hν* = 104 eV and at *T* = 35 K. which shows the two-fold symmetric FSs very clearly. Around Γ_1_, a smaller-size FS (the inner diamond) is stronger, while, around Γ_2_, a larger-size FS (the outer diamond) is stronger. (**b**) The candidate CDW-supercell structure having the 7 × 1 unit cell in the Te(2)-Te(3) sheets. Dumbells, connected by red bars, represent the dimerized Te(2)-Te(3) atoms in the *ab* plane. Trimers and tetramers are seen to be the basic building blocks. (**c**) The calculated FS that is obtained from the tight-binding (TB) model for the CDW-distorted 7 × 1 supercell structure shown in (**b**) and unfolded into the 2D-BZ. (**d**) The measured FS map of PrTe_2_ is shown for comparison, which was obtained with *hν* = 110 eV (corresponding to the Γ-plane of PrTe_2_) and at *T* = 35 K.

**Figure 4:**
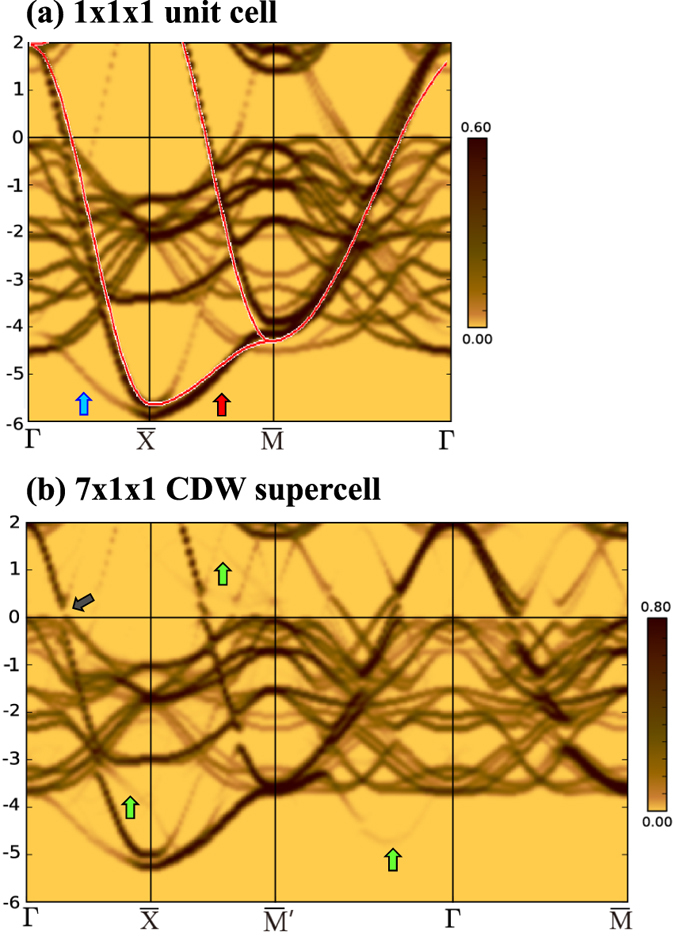
Calculated band structures of PrTe_3_ unfolded into the 2D-BZ. (**a**) Band structure of PrTe_3_ calculated for the 1 × 1 × 1 unit cell, and (**b**) Band structure of PrTe_3_ calculated for the 7 × 1 × 1 CDW-modulated supercell. 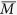 is along a^*^ (see Fig. 1(b)), while 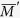 is along b^*^ that is perpendicular to the CDW vector *q*_*CDW*_. Red guide lines in (a) represent the TB band structure for the Te square net. The colored arrows indicate the main band (red), the 3D-folded shadow band (blue), the CDW-folded shadow band (green), and the CDW gap (black), respectively. To show the CDW gap more clearly, we considered the 20%-expanded volume of the 7 × 1 × 1 cell in (b).

**Figure 5:**
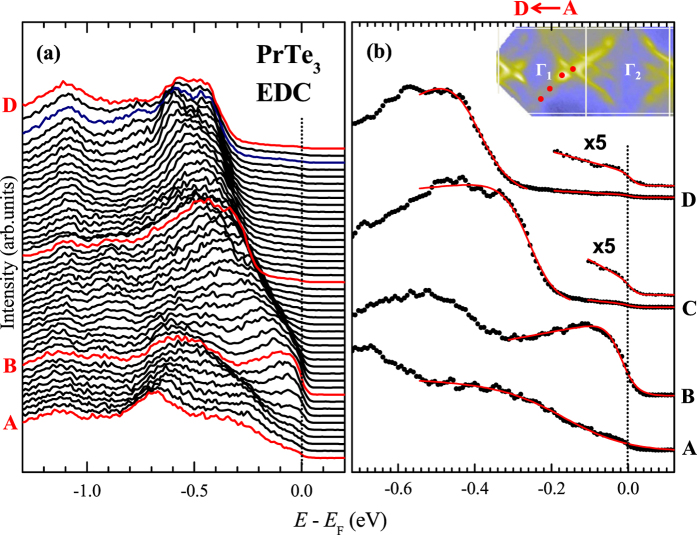
Near-E_F_ EDCs for PrTe_3_. (**a**) The near-E_F_ EDC stacks for PrTe_3_, obtained with *hν* = 104 eV and at *T* = 35 K. along the inner diamond-shaped FS (*A* → *D*), which is shown in the inset of (**b**) as the red dots. (**b**) Some EDCs selected from (**a**), on which guidelines (red lines) are superposed. For the EDCs obtained at *C* and *D*, the near-E_F_ regions are enlarged by ×5.

**Figure 6:**
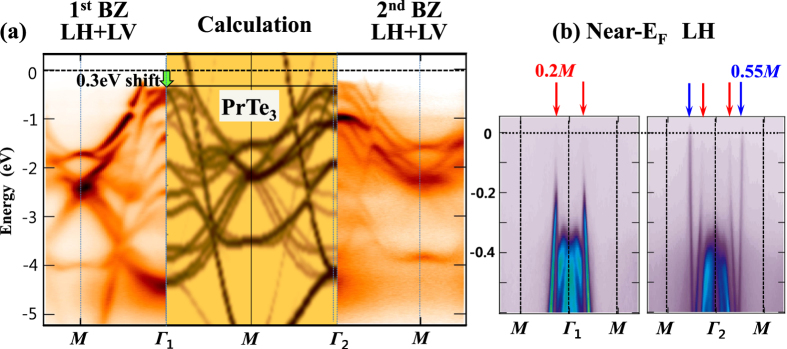
Comparison of the DFT-calculated band structures for the 7 × 1 × 1 CDW-modulated supercell of PrTe_3_ with ARPES (*T* = 35 K). The calculated bands are unfolded into the 2D-BZ. (**a**) The calculated band structures for the 7 × 1 × 1 CDW-modulated supercell of PrTe_3_ along Γ_1_*M*Γ_2_ (in the 3D-BZ notation of Fig. 1(b)), which are attached to the measured ARPES as mirror images. The ARPES image plots on the left and right sides are those for the first BZ and the second BZ, respectively. In the calculated band structures, the dark and light colors denote the main bands and the shadow bands (both the 3D-folded shadow bands and the CDW-folded shadow bands), respectively. ARPES data were obtained with *hν* = 104 eV and at *T* = 35 K. Nearly perfect agreement is obtained with the ∼0.3 eV shift of the calculated E_F_. (**b**) The measured ARPES image plots for the energy region near E_F_, for the first BZ (left) and for the second BZ (right). Two crossing bands are observed along Γ*M*, one near ∼0.2 Γ*M* (marked with red arrows) and the other at ∼0.55 Γ*M* (marked with blue arrows).

**Figure 7:**
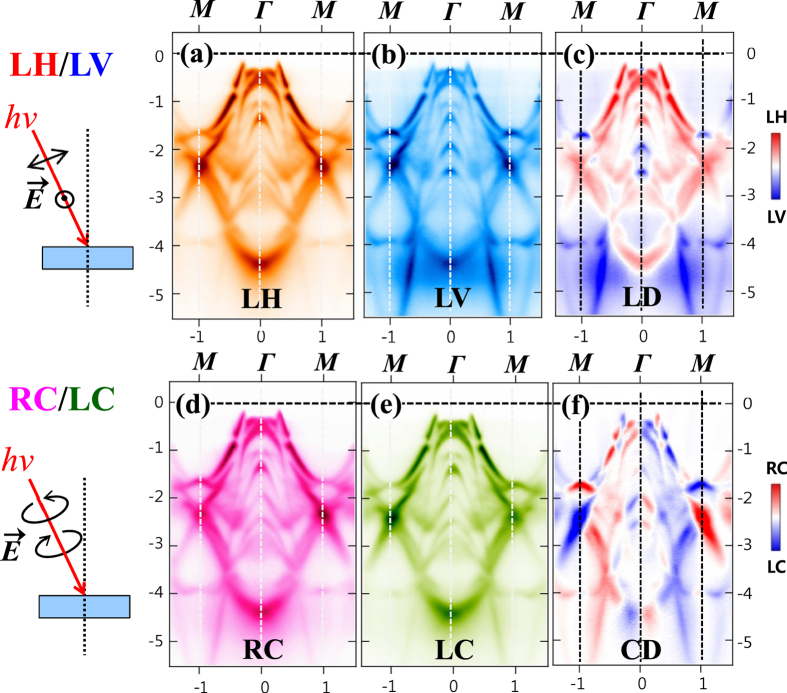
Linear Dichroism (LD) and Circular Dichroism (CD) in ARPES. (**a**–**c**) The measured ARPES image plots of PrTe_3_ along *M*Γ*M* (in the first BZ), obtained with the LH and LV polarization, and the difference (LD) between the LH and LV ARPES (LD ≡ LH-LV), respectively. (**d**–**f**) Similarly for the right circular (RC) and left circular (LC) polarization, and their differences (CD), respectively (CD ≡ RC-LC). These data were obtained with *hν* = 104 eV and at *T* = 35 K. The schematic drawings on the left show the configurations for LH/LV and RC/LC measurements. In the LD and CD ARPES plots ((**c**,**f**)), red color represents the stronger LH/RC intensity and blue color represents the stronger LV/LC intensity.

The electronic structure of PrTe_3_ has been investigated by employing ARPES, XAS, RPES, and first-principles band structure calculation, and the questions addressed in Introduction are answered. In particular, a possible candidate of the modulated structure of PrTe_3_ in the CDW phase has been identified Pr 3*d* XAS measurement provides evidence that the valence states of Pr ions are nearly trivalent (Pr^3+^) in PrTe_3_, supporting the previous consensus of the R-Te(1) layer being a charge reservoir, and the CDW instability in the partially-filled metallic Te(2)-Te(3) sheets. Pr 4*d* → 4 *f* RPES measurement reveals the finite Pr 4 *f* spectral intensity near E_F_, making a contrast to the negligible near-E_F_ intensity in PrTe_2_, which has one Te sheet only. This finding indicates the larger Pr 4 *f* contribution to the CDW formation in PrTe_3_ than that in PrTe_2_ through the Pr 4 *f*-Te(2)/Te(3) 5*p* hybridization. The two-fold symmetric FS has been clearly observed in PrTe_3_, which arises from the CDW-induced FS reconstruction. The CDW modulated structure in PrTe_3_ is likely to be the 7 × 1 structure (see Fig. 3(b)). The shadow bands and the corresponding very weak FSs are observed, originating from the band folding due to the interaction of Te(2)-Te(3) layers with Pr-Te(1) layers and also that due to the CDW-induced FS reconstruction. The effects of both LD and CD are observed in ARPES. The opposite effects of LD are observed between the E_F_-crossing states and the states at ∼1 eV BE. The E_F_-crossing bands exhibit the stronger intensity with the LV polarization than with the LH polarization, implying that the E_F_-crossing orbitals are ordered mainly in the *ab* plane, but not along the *c* axis. The observed CD in ARPES seems to reflect the existence of the experimental geometry effect, which, however, remains to be resolved theoretically. The straight vertical dispersions are observed along *k*_*z*_ in the *k*_*x*_ − *k*_*z*_ (*hν*) FS map, which demonstrates the 2D character for the near-E_F_ states in PrTe_3_. This finding implies the weak 3D interaction between Pr-Te(1) layer and Te(2)-Te(3) layers in PrTe_3_.

## Methods

### Sample Growth

High-quality PrTe_3_ single crystals were grown by using the self-fluxed Bridgeman method^46^. The quality and the orientation of the single crystal were checked by Laue patterns. PrTe_3_ has nearly the same lattice constants for the *ab* plane, but much larger lattice constant along the *c* axis due to the magnetic ordering along *c*: *a* = 4.375 \AA and *c* = 25.89 \AA^14^,^47^.

### ARPES and XAS Experiment

**Figure 8:**
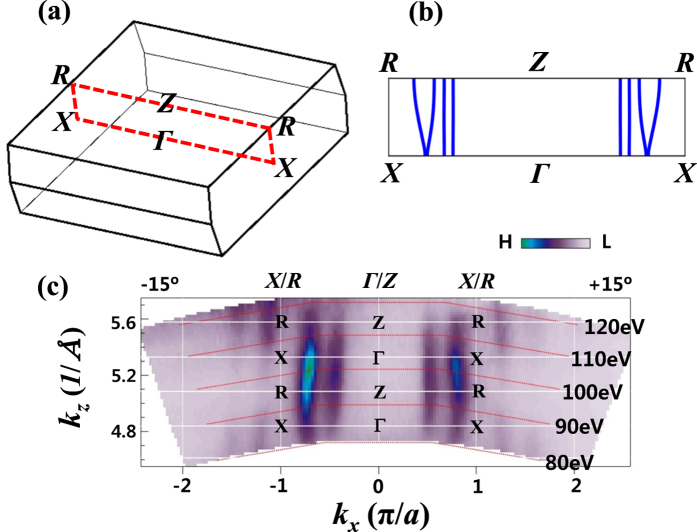
Photon Energy Map. (**a**) The *k*_*x*_ − *k*_*z*_ plane for which the *hν*-map was obtained in the BZ of PrTe_3_. The colored plane represents where the *hν*-map was obtained. (**b**) The calculated FS for the *k*_*x*_-*k*_*z*_ BZ in PrTe_3_. The 2D cylindrical FSs come from the in-plane *p* bands of Te(2) and Te(3) in the Te square net, while the outmost dispersive FS comes from the mixture of the out-of-plane *p* bands of Te(1) and the in-plane *p* bands of Te(2) and Te(3). (**c**) The Fermi-edge state *hν*-map for PrTe_3_, obtained for *hν* between 80–120 eV and at *T* = 35 K. The horizontal *k*_*x*_ direction is parallel to (100). This *hν*-map cuts through *X*Γ*X* (*RZR*) along *k*_*z*_ (*hν*).

ARPES measurements were carried out at the MERLIN beamline 4.0.3 at the Advanced Light Source (ALS). The ARPES endstation is equipped with a Scienta R8000 electron energy analyzer and a low temperature 6-axis sample manipulator cooled with an open-cycle He flow cryostat. Samples can be cooled down to 6 K, but we kept the samples at *T* ∼ 35 K to improve the electrical conductivity of them. Single crystalline samples were cleaved *in situ* and measured under the pressure better than 5 × 10^−11^ Torr. The photon energy (*hν*) corresponding to the Γ plane was determined from the photon energy scans for the Fermi-edge states at the normal emission^48^. For PrTe_3_, *hν* ≈ 104 eV turned out to be close to the Γ plane within the experimental uncertainty (see Fig. 8). The Fermi level (E_F_) and the instrumental resolution of the system were determined from the Fermi edge spectrum of an evaporated Au metal. The energy resolution of the data was set at ∼40 meV for *hν* = 104 eV. The momentum resolution (*k*-resolution Δ*k*) of the data was set to be less than Δ*k* ≤ 0.012 Å^−1^ in the detector angle. The *k*_*x*_ − *k*_*y*_ FS maps were obtained in the polar-compensation mode.

XAS experiment was performed at the 2A elliptically polarized undulator (EPU) beamline of the Pohang Light Source (PLS). XAS data were obtained by using the total electron yield (TEY) mode. Single crystalline samples were cleaved *in situ* and measured under the pressure better than 3 × 10^−10^ Torr and at *T* ∼ 80 K. The total resolution for XAS was set at ∼100 meV at *hv* ∼ 600 eV. All the spectra were normalized to the incident photon flux.

### DFT and Tight-Binding (TB) Model Calculations

For density functional theory (DFT) band calculations, we employed the *ab-initio* full-potential linearized augmented (FLAPW) band method implemented in Wien2k^49^. The pseudopotential band method implemented in VASP^50^ was also used for the band-unfolding^41^. The generalized-gradient approximation (GGA) was utilized for the exchange-correlation interaction, and the spin-orbit interaction was taken into account in the second variation manner.

For the TB model calculations, for simplicity, we have ignored the small corrugation of Te atoms in the Te bilayer, and considered the single Te layer only. The *p*_*z*_ orbitals are ignored because these orbitals are far from E_F_. The effect of the lattice distortion is ignored in this simple TB model. The TB hopping parameters of (*pp*_*σ*_ ; *pp*_*π*_) for the long and short bonds are taken as (1.4 ; −0.5) and (2.0 ; −0.3), respectively.

## Additional Information

**How to cite this article**: Lee, E. *et al*. The 7×1 Fermi Surface Reconstruction in a Two-dimensional *f*-electron Charge Density Wave System: PrTe_3_. *Sci. Rep.* **6**, 30318; doi: 10.1038/srep30318 (2016).

## Supplementary Material

Supplementary Information
