# Supplementary material for: The 7 × 1 Fermi Surface Reconstruction in a Two-dimensional f -electron Charge Density Wave System: PrTe3
Source: Sci Rep. 2016 Jul 25;6:30318. doi: 10.1038/srep30318 (PMC4958976; doi:10.1038/srep30318)
Supplement: Supplementary Information [file srep30318-s1.pdf]

Supplementary Information

The 7×1 Fermi Surface Reconstruction in a Two-dimensional f-electron Charge Density Wave System: $PrTe_3$

Eunsook Lee[1], D. H. Kim[1], Hyun Woo Kim[1], J. D. Denlinger[2], Heejung Kim[3], Junwon Kim[3], Kyoo Kim[3,4], B. I. Min[3], B. H. Min[5],[*] Y. S. Kwon[5], and J.-S. Kang[1†]

[1]*Department of Physics, The Catholic University of Korea, Bucheon 14662, Korea*

[2]*Advanced Light Source (ALS), Lawrence Berkeley Laboratory, Berkeley, CA. 12345, USA*

[3]*Department of Physics, Pohang University of Science and Technology, Pohang 37673, Korea*

[4]*MPPC_CPM, Pohang University of Science and Technology, Pohang 37673, Korea and*

[5]*Department of Emerging Materials Science, DGIST, Daegu 42988, Korea*

FERMI SURFACE AND CONSTANT-ENERGY MAPS OF PRTE$_3$

Figure S1(a) shows the Fermi Surface (FS) and constant-energy (CE) slices of the angle maps of $PrTe_3$ from E_F ($E_F \equiv 0$ eV: FS map) to BE=1.0 eV (BE=$|E_i|$, where BE and E_i denote the binding energy and the initial-state energy, respectively). These angle-map data were obtained in the polar-compensation mode. Then, in constructing the FS/CE intensity maps, no further artificial normalization was done for the spectral intensity distribution of (k_x, k_y). The horizontal direction is along (110), and the FS/CE maps in this figure were made by integrating the spectral intensity of $E_i \pm 40$ meV for each E_i. The overall intensity of each map has been scaled by an appropriate scale factor, as described in the figure caption. Notable is that the nearly four-fold symmetry is observed in the CE maps for BE$\geq$ 0.3 eV, while the clear two-fold mirror symmetry is observed in the FS.

Figure S1(b) shows the calculated FS and CE maps for $PrTe_3$ in the non-CDW phase, obtained from the density functional theory (DFT) band calculations. Reasonable agreement is found between the measured CE maps (Fig. S1(a)) and the calculated CE maps for an ideal Te square net (Fig. S1(b)). A close look, however, shows a disagreement between the measured FS and the calculated FS that exhibits the four-fold symmetry. Such a disagreement comes from the fact that the calculated FS is obtained for the non-CDW phase of $PrTe_3$, the structure of which is almost tetragonal. Such a two-fold symmetric FS of $PrTe_3$ makes a contrast with the four-fold symmetric FS of $PrTe_2$, which is shown in Fig. 3(d) in the main text.

THE RELAXED ATOMIC POSITIONS OF TE IN THE $7 \times 1 \times 1$ SUPERCELL STRUCTURE

As for the relaxation calculation, we first assumed local distortions, as presented in Fig. 3(b) in the main text, and then let the system relax around this assumed structure. We have found that such local distortions are stable, indicating that the distorted structure is at least metastable. Figure S2 shows the relaxed positions of Te atoms in the 7-fold supercell. Here red points represent the atomic positions of Te atoms. Interestingly, it is seen that the relaxed atomic positions maintaining oligomer patterns are described qualitatively well by the sinusoidal function with $q = 2/7\ a^*$ (blue-dotted line). This result shows that the CDW structure of $PrTe_3$ can be described by either oligomers or sinusoidal distortions. This finding supports that the $7 \times 1 \times 1$ supercell structure is close to the correct CDW structure of $PrTe_3$.

---

[*] Present address: CeNSCMR, Department of Physics and Astronomy, Seoul National University, Seoul 08826, Korea

[†] kangjs@catholic.ac.kr

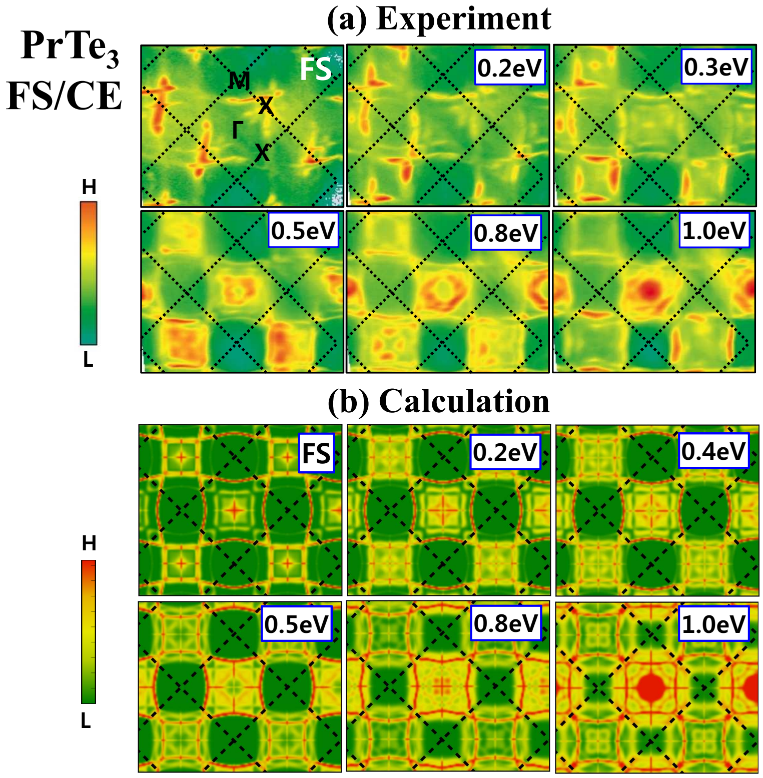

FIG. S1. (Color online) The measured and DFT-calculated FS and CE maps for $PrTe_3$. (a) The constant-energy (CE) maps of the ARPES intensity in the momentum space at increasing binding energies (BEs) for $PrTe_3$ from BE=0 eV (E_F) to BE=1.0 eV (BE=$|E_i|$). The horizontal direction is along (110). These data were obtained with $h\nu = 104$ eV and at $T = 35$ K. Dotted lines denote the 3D-BZ. In (a), the overall intensity of each FS/CE map is scaled with respect to that of $|E_i|$=1.0 eV (scale factor=1). The intensities of FS/CE maps for $|E_i|$=0 (FS), 0.2, 0.3, 0.5, and 0.8 eV are multiplied by 22, 5, 3.6, 1,3, and 1.3, respectively. (b) The calculated FS and CE maps for the undistorted non-CDW phase of $PrTe_3$, which are unfolded into 2D-BZ. Here the center corresponds to Γ_2 (the Γ point in the second BZ).

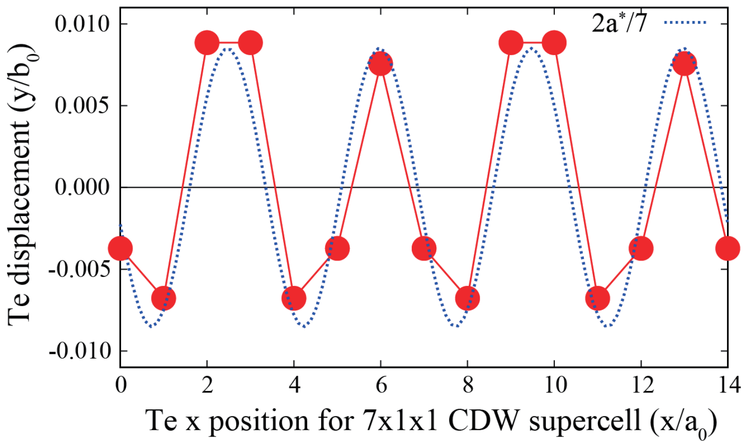

FIG. S2. (Color online) Relaxed positions of Te atoms in the 7-fold supercell (red points). Blue-dotted lines represent the sinusoidal function with $q = 2/7\ a^*$, which describes the distorted Te positions qualitatively well. Here a_0 and b_0 are lattice constants of 1×1 $PrTe_3$.
